# Reversible cerebral vasoconstriction syndrome presenting as an isolated primary intraventricular hemorrhage

**DOI:** 10.1186/s41016-018-0118-7

**Published:** 2018-06-04

**Authors:** Katarina Dakay, Ryan A. McTaggart, Mahesh V. Jayaraman, Shadi Yaghi, Linda C. Wendell

**Affiliations:** 1 Department of Neurology, Rhode Island Hospital/The Warren Alpert Medical School of Brown University, Providence, RI USA; 2 Departments of Radiology, Neurology, and Neurosurgery, Rhode Island Hospital/The Warren Alpert Medical School of Brown University, Providence, RI USA; 3 Department of Neurology, Neurosurgery and Medical Education, Rhode Island Hospital/The Warren Alpert Medical School of Brown University, 593 Eddy St APC 712, Providence, RI 02903 USA

**Keywords:** Reversible cerebral vasoconstriction syndrome, Pseudoephedrine, Primary intraventricular hemorrhage

## Abstract

**Background:**

Primary intraventricular hemorrhage is an uncommon cause of stroke and is often associated with longstanding, uncontrolled hypertension. Reversible cerebral vasoconstriction is also an uncommon condition characterized by reversible constriction of intracerebral vessels, which can lead to ischemic or hemorrhagic strokes.

**Case presentation:**

We describe a case of isolated primary intraventricular hemorrhage secondary to reversible cerebral vasoconstriction syndrome triggered by pseudoephedrine.

**Conclusions:**

Reversible cerebral vasoconstriction syndrome is a rare cause of primary intraventricular hemorrhage and should be considered in the differential in angiography-negative IVH when there is a history of vasoactive substance use.

## Background

Reversible cerebral vasoconstriction syndrome (RCVS) is an uncommon condition of reversible vasospasm of intracerebral vessels which manifests as a thunderclap headache, focal neurologic deficits and often both ischemic and hemorrhagic strokes including subarachnoid hemorrhage. It is felt to be secondary to disturbances in vascular tone, and is often triggered by use of vasoactive substances, though it may also be seen in the postpartum setting [1]. High cortical subarachnoid hemorrhages are commonly reported in RCVS. However, primary intraventricular hemorrhage has not previously been described as a hemorrhagic manifestation of RCVS. Primary intraventricular hemorrhage (IVH) is a rare cause of intracerebral hemorrhage; it accounts for 3.1% of hemorrhagic strokes [2] and 0.31% of all strokes [3]. IVH has only once been previously described in the literature with regards to reversible cerebral vasoconstriction and occurred in context of additional ischemic lesions rather than as an isolated manifestation [4]. To our knowledge, this is the first case reported of RCVS causing isolated intraventricular hemorrhage.

## Case presentation

**Fig. 1:**
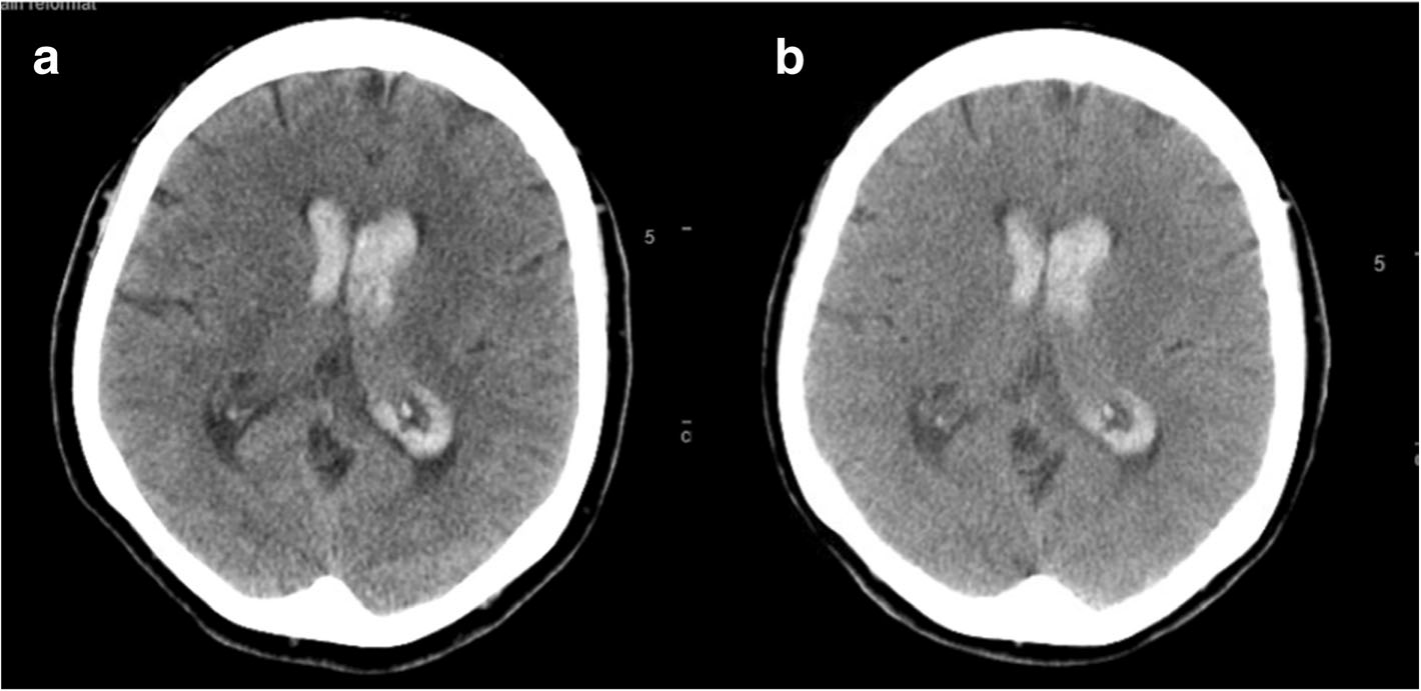
**:** Noncontrast head CT demonstrated intraventricular hemorrhage (IVH) **a** Initial head CT showing diffuse IVH in the bilateral lateral ventricles. **b** Follow-up head CT showing stable IVH

A 58 year-old woman presented to an outside hospital after developing the “worst headache of her life” followed by abrupt collapse with convulsive movements. At the outside hospital, a noncontrast head CT demonstrated IVH (Fig. 1a). Her mental status then deteriorated, requiring emergent intubation, and she was transferred to our hospital.

Her past medical history included chronic migraines, depression and seasonal allergies. She had no prior surgeries. Her home medications included two antidepressants with serotonergic activity, bupropion and sertraline. In the past few weeks prior to presentation, she had been using an over-the-counter decongestant on a daily basis which contained pseudoephedrine. She had no history of autoimmune disease, vasculitis, hypertension or stroke. She did not endorse any history of illicit or recreational drug use.

Upon arrival to our hospital, she was hypertensive with a systolic blood pressure of 200 mmHg despite no previous history of hypertension. Her lab studies on admission included a negative toxicology screen which excluded presence of amphetamines or cocaine. Platelet count and coagulation parameters were within normal limits. Electrocardiogram demonstrated sinus rhythm.

**Fig. 2:**
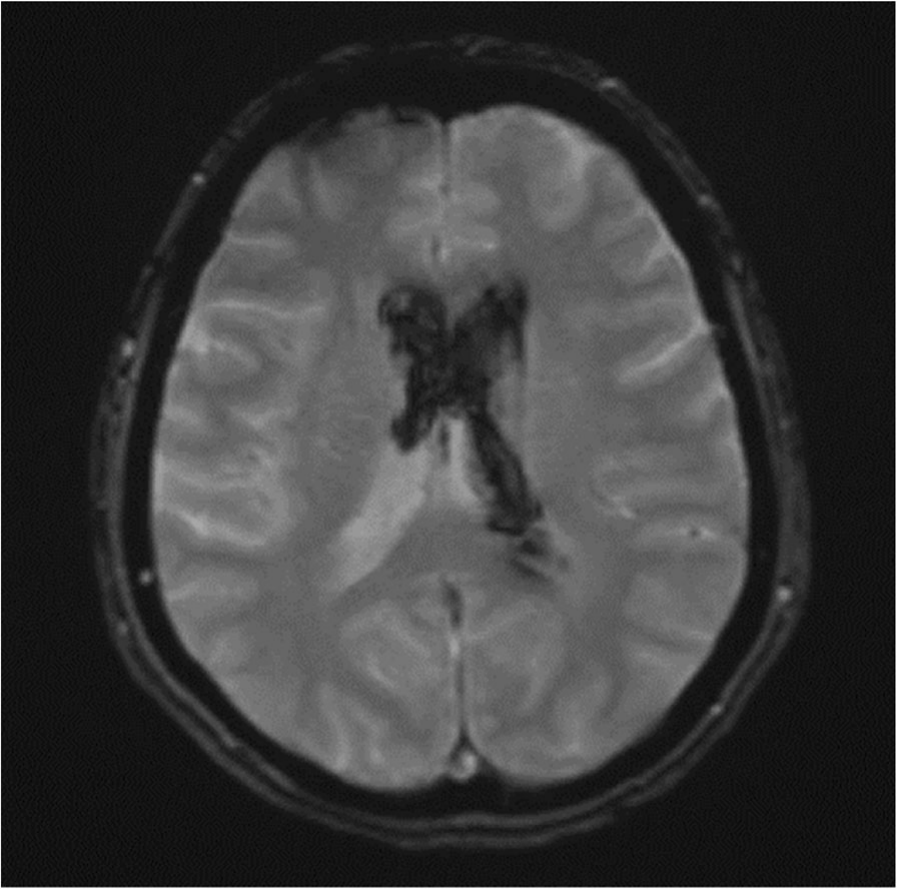
MRI brain [fast low angle shot (FLASH) sequence] demonstrated intraventricular hemorrhage; no causative underlying vascular lesion was identified

**Fig. 3:**
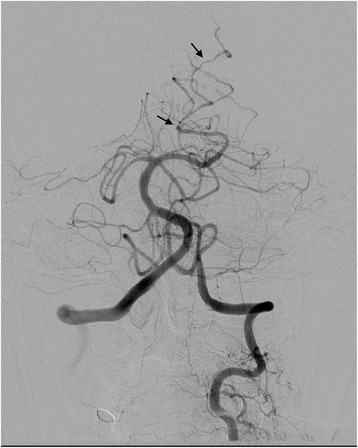
Catheter angiography of left vertebral artery demonstrated vasoconstriction. The arrows point to multifocal areas of irregular narrowing of the distal branches of the left posterior cerebral artery

The patient’s blood pressure was treated with a continuous infusion of nicardipine with a goal systolic blood pressure less than 160 mmHg, and she was admitted to the neurocritical care unit. Repeat noncontrast head CT 6 hours after her initial head CT was unchanged (Fig. 1b). CT angiogram of the brain did not demonstrate an underlying vascular abnormality. The patient was monitored for risk of hydrocephalus, which did not develop. An MRI of the brain performed on the fourth day of admission redemonstrated the IVH without any intraparenchymal component or underlying vascular malformation (Fig. 2). Transcranial Doppler on hospital days five and nine demonstrated normal velocities. Subsequently, conventional catheter angiography on day five of admission was obtained which showed multifocal areas of irregular narrowing in the distal posterior cerebral artery branches and distal left middle cerebral artery consistent with a vasculopathy (Fig. 3).

The patient improved clinically and was extubated; however, she continued to have a severe headache. Oral verapamil resulted in mild improvement. Subsequently, 2 g of intravenous magnesium were administered followed by oral magnesium gluconate; the patient reported a dramatic improvement in her headache and was discharged.

The diagnosis on the basis of the history of vasoactive substance use, severe acute headache, and characteristic angiography findings was a primary IVH secondary to RCVS. The trigger was felt to be pseudoephedrine with possible contributing factors being the use of bupropion and sertraline, leading to altered vascular tone. Vasculitis was felt to be unlikely given the abrupt onset of symptoms and rapid clinical improvement. Bupropion, sertraline and pseudoephedrine were held throughout her hospitalization and discontinued upon discharge. The patient presented to clinic follow-up 2 months later and reported resolution of symptoms; thus, follow-up vascular imaging was deferred. She has not had any recurrence of thunderclap headaches since discharge.

## Discussion and conclusions

RCVS is a clinical syndrome characterized by acute thunderclap headache with nausea and vomiting often mimicking aneurysmal subarachnoid hemorrhage. It is more common in women than in men [1]. Its defining features include findings of segmental vasoconstriction on angiography, lack of aneurysmal source, normal or near normal cerebrospinal fluid, thunderclap headache, and reversibility of the lesion [5]. It is felt to be due to alterations in vascular tone and likely affects the distal vasculature primarily, progressing more proximally towards the vessels of the Circle of Willis [6]. These changes are often not seen on noninvasive vessel imaging. Likewise, in our case, a CT angiogram of the brain did not show the typical diffuse stenoses and dilatation of vessels characteristic of RCVS, but these findings were very apparent on catheter angiography.

RCVS is often triggered by vasoactive substances including selective serotonin reuptake inhibitors and sympathomimetic medications; illicit substances including cocaine, ecstasy, and marijuana have also been implicated [1]. In our patient, pseudoephedrine, which has previously been described as a precipitant, was felt to be the primary causative factor given the temporal relationship of the usage to the development of symptoms. However, bupropion and sertraline both have serotonin reuptake inhibition activity and were also identified as possible precipitating factors. The mechanism by which sympathomimetic overactivity leads to RCVS is not known, however, one theory is that genetically susceptible patients may develop microvascular inflammation in response to sympathetic overstimulation, leading to disruption in arteriolar tone [7]. It is important to note that sympathomimetics including pseudoephedrine, phenylephrine, and oxymetazoline are commonly available over-the-counter without a prescription and used to treat allergies, sinus congestion, and occasionally epistaxis.

Primary IVH is a rare cause of intracerebral hemorrhage and typically presents either with abrupt sudden coma followed by signs of brainstem dysfunction or a waxing and waning headache followed by nausea, vomiting and a progressive confusional state. One striking feature differentiating primary IVH from other types of intracerebral hemorrhage is either a lack of, or very minimal, focal neurologic deficits. Hypertension is a common risk factor for IVH. However, angiography is recommended given the potential for underlying vascular malformations.

In the discussed case, angiography was undertaken given the risk of an underlying causative vascular malformation, which has been reported to be as high as 56% when combining various case studies [8]. However, catheter angiography yielded no evidence of aneurysm or arteriovenous malformation, but instead demonstrated beading and dilatation of the distal vessels suggestive of vasculopathy. Given the rapid improvement in clinical status after withdrawal of the offending substances and initiation of calcium channel blocker medication as well as magnesium, the overall clinical picture supported a diagnosis of RCVS.

Though head CT can be normal in RCVS, common imaging abnormalities seen include ischemic stroke, high cortical subarachnoid hemorrhage, vasogenic edema, and lobar hemorrhage [1, 9]. One large case series reported that 43% of patients with RCVS have hemorrhagic complications [10]. Subdural hemorrhage is rarely seen but has been reported. Two previous case studies have reported IVH in the setting of RCVS [4, 11], indicating that it is a rare phenomenon. In one case, an patient with RCVS triggered by phenylephrine developed an intraparenchymal hematoma which extended into the subarachnoid and intraventricular spaces [11]; however, in this situation, the IVH was secondary to a primary intraparenchymal hematoma. A second case demonstrated a primary IVH secondary to reversible cerebral vasoconstriction which occurred in the context of the nasal decongestant oxymetazoline; in this situation prepontine cisternal and fourth ventricular hemorrhages were demonstrated, but occurred in the context of multifocal ischemic strokes more typical of that seen in RCVS [4]. The mechanism by which RCVS leads to hemorrhagic complications, including intraventricular hemorrhage is not well-elucidated; however, it is postulated that rapid changes in vascular caliber due to vasoconstriction and subsequent vasodilatation can lead to reperfusion injury and subsequent hemorrhage [10]. To our knowledge, our case is the first of an isolated primary IVH without additional lesions.

Treatment of RCVS involves withdrawal of the offending substance and supportive care. While there are no randomized controlled trials supporting the efficacy of calcium channel blockers, several case series have reported improvement in symptoms with usage of these agents and they are currently recommended on this basis [5]. Steroids were associated with a trend towards poor outcome in one two-center case series though interpretation is limited as the series was retrospective. The outcome of RCVS is generally favorable [1], though hemorrhage increases the risk of disability [9]. Fortunately, the patient described in our case was asymptomatic at follow-up several weeks after discharge.

RCVS may be a potential culprit in cases of primary IVH in which a causative aneurysm or arteriovenous malformation cannot be identified. Our case study was limited in the sense that the patient did not have follow-up imaging, as the additional radiation was felt to be unnecessary due to the complete resolution of symptoms. It is also not possible to prove the sequence of the vasoconstriction and the hemorrhage, as vessel imaging was obtained only after the patient was found to have a hemorrhage, although the diffuse nature of the vasoconstriction was felt to more likely represent the cause of the intraventricular hemorrhage rather than an effect. However, the important point in this case is the potential serious consequences of vasoactive substances such as over-the-counter decongestants, which have been associated with triggering RCVS. Because these substances are readily available without a prescription, it is important to inquire about the use of these substances, as prompt identification and withdrawal of the offending agent reduces the risk of further neurologic decline.

## Abbreviations

RCVS: Reversible cerebral vasoconstriction syndrome
IVH: Intraventricular hemorrhage
CT: Computerized tomography
MRI: Magnetic resonance imaging
